# Supplementary material for: One versus two doses of ivermectin-based mass drug administration for the control of scabies: A cluster randomised non-inferiority trial
Source: PLoS Negl Trop Dis. 2023 Mar 17;17(3):e0011207. doi: 10.1371/journal.pntd.0011207 (PMC10058081; doi:10.1371/journal.pntd.0011207)
Supplement: S1 Table — (DOCX) [file pntd.0011207.s001.docx]

**Table S1. Scabies prevalence at 21 months by demographic group according to self-reported participation in mass drug administration**

|  | Arm 1 (1 dose MDA) |  | Arm 2 (2 dose MDA) | |
| --- | --- | --- | --- | --- |
|  | **Participated at baseline**  **N (%, 95% CI)** | **Did not participate at baseline**  **N (%, 95% CI)** | **Participated at baseline**  **N (%, 95% CI)** | **Did not participate at baseline**  **N (%, 95% CI)** |
| Total participants | 804 (47·8) | 877 (52·2) | 1057 (63·0) | 622 (37·0) |
| Scabies | 128 (15·9, 13·5-18·6) | 186 (21·2, 18·5-24·1) | 114 (10·8, 9·0-12·8) | 111 (17·8, 14·9-21·1) |
| Sex |  |  |  |  |
| Female | 69 (14·6, 11·5-18·1) | 79 (15·6, 12·5-19·0) | 55 (8·7, 6·6-11·2) | 61 (18·0, 14·1-22·6) |
| Male | 59 (17·9, 13·9-22·4) | 107 (28·9, 24·3-33·8) | 59 (13·8, 10·7-17·5) | 50 (17·6, 13·4-22·5) |
| Age (years) |  |  |  |  |
| 0-1 | 0 | 23 (34·3, 23·2-46·9) | 0 | 22 (30·6, 20·2-42·5) |
| 2-4 | 16 (34·8, 21·4-50·2) | 24 (28·6, 19·2-39·5) | 10 (17·2, 8·6-29·4) | 28 (33·3, 23·4-44·5) |
| 5-9 | 49 (25·4, 19·4-32·1) | 43 (28·9, 21·7-36·8) | 44 (16·1, 12·0-21·0) | 16 (20·5, 12·2-31·2) |
| 10-14 | 28 (15·6, 10·6-21·7) | 46 (21·8, 16·4-28·0) | 38 (17·0, 12·3-22·5) | 17 (14·9, 8·9-22·8) |
| 15-19 | 4 (10·3, 28·7-24·2) | 13 (11·9, 6·5-19·5) | 2 (3·3, 0·4-11·3) | 4 (4·6, 1·3-11·4) |
| 20-29 | 9 (15·6, 7·3-27·4) | 9 (10·7, 5·0-19·4) | 6 (7·0, 2·6-14·6) | 6 (12·2, 4·6-24·8) |
| 30-39 | 7 (8·1, 3·3-16·1) | 8 (11·6, 5·1-21·6) | 4 (3·6, 1·0-9·0) | 8 (13·6, 6·0-25·0) |
| 40-49 | 6 (8·3, 3·1-17·3) | 3 (7·0- 1·5-19·1) | 5 (5·1, 1·7-11·4) | 5 (14·3, 4·8-30·3) |
| 50-59 | 6 (10·0, 3·8-20·5) | 10 (25·6, 13·0-42·1) | 1 (1·4, 0-7·6) | 2 (8·7, 1·1-28·0) |
| 60+ | 3 (4·3, 0·9-12·0) | 7 (31·8, 13·9-54·9) | 4 (5·3, 1·5-13·1) | 3 (14·3, 3·0-36·3) |

MDA – mass drug administration

95% CI – 95% confidence interval
