# Supplementary material for: One versus two doses of ivermectin-based mass drug administration for the control of scabies: A cluster randomised non-inferiority trial
Source: PLoS Negl Trop Dis. 2023 Mar 17;17(3):e0011207. doi: 10.1371/journal.pntd.0011207 (PMC10058081; doi:10.1371/journal.pntd.0011207)
Supplement: Attachment — Submitted filename: Response to reviewers .docx [file pntd.0011207.s003.docx]

23rd January 2023

The Editor

PLOS Neglected Tropical Diseases

**Manuscript ID: PNTD-D-22-01304**

**Title: One versus two doses of ivermectin-based mass drug administration for the control of scabies: A cluster randomised non-inferiority trial**

Thank you for providing us with reviewer comments on our paper. Please find our point-by-point response below.

We have submitted two copies of the revision, one with tracked changes and the other a clean copy.

We look forward to hearing back from you.

On behalf of all authors, yours sincerely,

**Susanna Lake**, Murdoch Children’s Research Institute

**John Kaldor**, The Kirby Institute, UNSW Sydney

**Editor’s comments**

***1. Line 362. needs rewording***

We have reworded this line to: Movement of people in and out of study villages may have re-introduced scabies to these communities.

**Methods**

**Reviewer 1:**

***2. In writing up the study both in the abstract and conclusions the authors should make the exceptional circumstances clearer together with lessons that can be learned. Reintroduction of scabies into mainland treated communities by untreated patients has already been highlighted as a public health challenge. This study should have provided the opportunity to emphasise this. Otherwise it simply reads as a well conducted but failed experiment. Some rewording is required***

The Abstract. Line 83-88 now read: ‘There was no indication of an overall decline in scabies prevalence in either arm. The reduction in scabies prevalence in those present at baseline suggests that the unexpectedly high influx of people into the trial villages, likely related to the COVID-19 pandemic, may have compromised the effectiveness of the MDA. Despite the lack of effect there are important lessons to be learnt from this trial about conducting MDA for scabies in high prevalence settings.’

We have added the following text to the Discussion at line 379-382: ‘An important lesson from this trial may be that in settings with very high prevalence of scabies, MDA needs to go beyond individual villages, to encompass whole populations that have interlinked connections via school, work and other forms of engagement.’

We also added at line 408-410: ‘Despite the exceptional circumstances faced by the RISE trial, our observations can serve to inform the design of scabies control programs and trials of MDA strategies.’

**Results**

**Reviewer 1:**

***3. Please provide a reference for the case definition of impetigo used .***

We have added a reference (line 185).

***4. Can you provide details of the permethrin treatment regimen followed ?***

We have added to the information on the permethrin treatment regimen already provided in line 167-170, with new text in line 171-173: ‘The dosing regimen for permethrin was the same as that for ivermectin and determined by the village allocation; either one dose (Arm 1) or two doses 7-14 days apart (Arm 2).’

***5. Can you comment on the changes in impetigo data – do they provide any support for the view that population movements accounted for the unexpected results recorded.***

We have added comment on this to the Discussion in line 350-352: ‘These new cases introduced to the participating communities may have been at a milder clinical stage of scabies infestation, thereby providing an explanation for both the reduced scabies severity and the reduced impetigo prevalence.’

**Conclusions**

**Reviewer 1:**

***6. In other studies how significant has been failure to recall receipt of MDA treatment by patients as a factor in producing discordant results?***

There is limited information about the accuracy of recall of MDA after 12 months. We have added detail about this in line 361-363 along with a reference: ‘There is evidence that people can accurately recall whether or not they have received MDA up to 12 months after treatment, there is not published information on recall accuracy at longer time intervals.’

***7. What lessons does this work this provide for future studies or strategies for scabies control teams?***

See response to point 2 above.

**Editorial and data presentation modifications**

**Reviewer 2:**

***8. Page 13 line 264 - do you really need both table 2 and figure 3? I think there might be too many tables so suggest considering leaving out 2***

We have removed table 2 and added it to the Supplementary Data.

***9. Page 16 line 300 you mention impetigo is reduced post MDA etc referencing table 2 - I think you mean table 3.***

We have corrected this reference.
